# The Eye Pupil Adjusts to Illusorily Expanding Holes

**DOI:** 10.3389/fnhum.2022.877249

**Published:** 2022-05-30

**Authors:** Bruno Laeng, Shoaib Nabil, Akiyoshi Kitaoka

**Affiliations:** ^1^Department of Psychology, University of Oslo, Oslo, Norway; ^2^Department of Psychology, Ritsumeikan University, Osaka, Japan

**Keywords:** illusory motion, pupillometry, brightness, color, perceiving-the-present

## Abstract

Some static patterns evoke the perception of an illusory expanding central region or “hole.” We asked observers to rate the magnitudes of illusory motion or expansion of black holes, and these predicted the degree of dilation of the pupil, measured with an eye tracker. In contrast, when the “holes” were colored (including white), i.e., emitted light, these patterns constricted the pupils, but the subjective expansions were also weaker compared with the black holes. The change rates of pupil diameters were significantly related to the illusory motion phenomenology only with the black holes. These findings can be accounted for within a perceiving-the-present account of visual illusions, where both the illusory motion and the pupillary adjustments represent compensatory mechanisms to the perception of the next moment, based on shared experiences with the ecological regularities of light.

## Introduction

**FIGURE 1:**
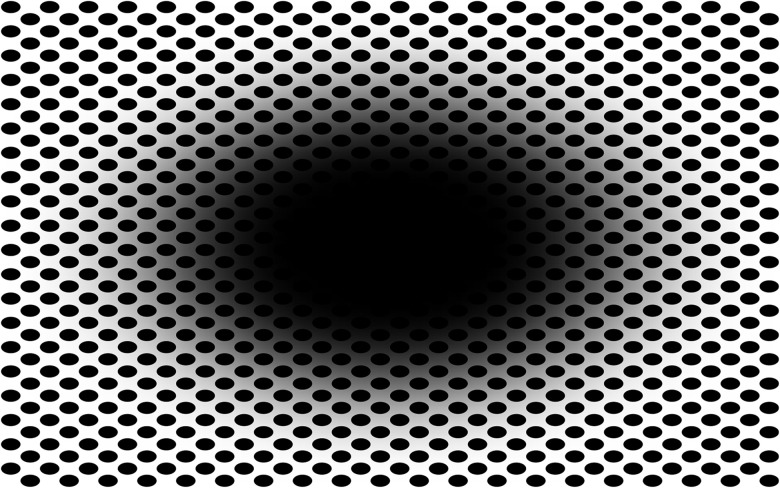
Example of illusorily expanding central region or “hole.” (Nota Bene: viewing the image full screen is best for eliciting the effect).

A large class of optical patterns evoke conscious dynamic sensations of illusory movement, despite being static. These illusory motions can be described as a variety of changes in shape or space, like drifting, rotating, oscillating, waving, fluttering, contracting, or expanding. An example of this type of illusion, which we call “expanding hole” is illustrated in Figure 1. Typically, when looking at the pattern below, observers’ subjective reports are characterized by the perception of a gradually expanding central region, occurring over a span of several seconds. According to several reviews of visual illusions (Coren et al., 1976; Changizi and Widders, 2002; Changizi et al., 2008; Wade, 2014), illusions of extent or size are a prominent class (e.g., Ponzo, Müller-Lyer, Ebbinghaus, etc.). However, classic illusions of size do not evoke dynamic sensations of motion like the “expanding hole” presented here.

Given that the above type of illusion also implies an illusory change in luminance, since it implies expanding darkness, it is possible to probe the impact of the illusion not only *via* an observer’s phenomenology, consisting of conscious verbal reports, but also by examining a concurrent, involuntary, physiological index: the diameter of the eye pupil.

Such a two-pronged approach, uniting phenomenology with psychophysiology, has been used previously with other illusions of brightness, showing spontaneous pupil adjustments to illusions of luminosity from gradient patterns (e.g., Laeng and Endestad, 2012; Binda et al., 2013; Naber and Nakayama, 2013; Zavagno et al., 2017; Suzuki et al., 2019; Sulutvedt et al., 2021).

**FIGURE 2:**
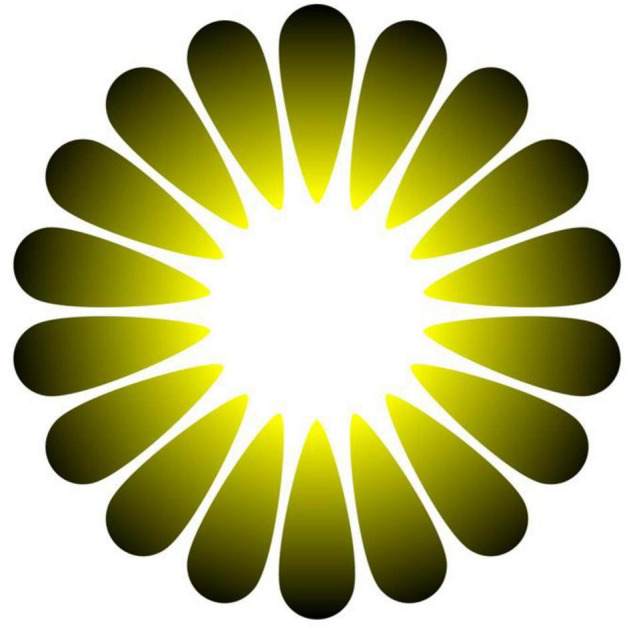
The “Asahi” illusion of brightness by Akiyoshi Kitaoka. The central region looks more luminous than the white background surrounding the gradients though it is the same white everywhere.

At the root of these bright/dark illusions is the fact that, in general, the perception of light is not straightforwardly related to physical parameters (Purves et al., 2004, 2014); hence, the visual system relies on ecological regularities or constraints to generate perceptual hypotheses that, in most instances, achieve pragmatically the behavioral success of vision (Purves et al., 2011). In the case of the ‘‘Asahi’’ illusion,^1^ we surmise that the visual system is prepared from individual experience (or that of the species) with the natural statistics of the visual world (Long et al., 2006), to the specific ecological condition of seeing sunlight through leaves or clouds (Zavagno et al., 2017; Suzuki et al., 2019). In fact, brightness illusions like Asahi (Figure 2) bear a geometric resemblance to the gradients shaped by the glare of strong sunlight when partially occluded by plant leaves or cloud formations.

Although the psychophysiological adjustments of the eye pupil to illusory patterns may seem useless, according to this perspective, these changes in diameter could be more adaptive than pupil responses based on a more precise scaling of the physical features of visual stimuli. Specifically, a preparatory constriction to illusory light could have an important function by promoting behavior to avoid being “dazzled” by physical (real) light. In fact, glare can temporarily incapacitate sight and lead to life-threatening situations (e.g., it is a common cause of lethal traffic accidents; Gao and Pei, 2009). In general, by constricting its diameter, the pupil may moderate the amount of energy entering the eye, not only to optimize vision but also to protect the retina from excessive light exposure. Radiant energy of electromagnetic radiations from both natural and artificial light sources can lead to injuries, if strong or prolonged, to the retina or other ocular structures through photothermal, photochemical, and photomechanical mechanisms (Contín et al., 2016).

In this case, the illusory motion of the dark central region in Figure 1 evokes the opposite illusory effect of the white central region in Figure 2. Observers experience a decrease in perceived brightness or a growing sense of darkness, as if entering a space voided of light. According to a theoretical perspective called “perceiving-the-present” (Changizi et al., 2008), illusions of dynamic shape or spatial change are useful because they generate the perception of the likely outcome of a forward motion by the viewer or the way the world will look about 100 milliseconds (ms) later, that is, perception during motion involves a compensation of the neural delay of visual information from the retina to cortical areas supporting visual consciousness. This seems even necessary because, at the time retinal information reaches higher areas, either the world or the observer’s position has changed (Changizi and Widders, 2002). In the specific case of the expanding hole illusion, we suggested that the circular smear or shadow gradient of the central black hole evokes a marked impression of optic flow, as if the observer were heading forward into a hole or tunnel, that is, (a) “smear” cues in the stimulus suggest a probable forward direction of motion and (b) the visual neural compensation network predicts how the stimulus would change in the next moment and generates an illusory “outward expansion” of the central “hole” region.

In other words, pupil changes to illusorily expanding stimuli might obtain the benefit of preparing the visual system, already at the input level, to a change in luminance in the next, predicted, moment and according to a fundamentally probabilistic strategy of vision. Just as glare can dazzle, being plunged into darkness is likely risky when navigating into the darkened environment (e.g., by possible collisions into objects and/or instability on uneven grounds). Although, as in any illusion, this virtual expanding darkness is experienced at the cost of veridicality, since the observer is neither moving forward nor entering any dark space, such a cost is likely to be less severe than if there were no corrections when an observer really moved forward into a dark space (Changizi et al., 2008). Hence, a pupil adjustment to the dynamic illusion would suggest optimal adaptation to an ecological regularity, instead of an “inappropriate” perceptual strategy or a failure in information processing.

In this study, we showed several “hole” patterns for a few seconds on a computer screen while, using an infrared eye tracker, we recorded the concurrent adjustments of the eye pupil to the perceived illusory expansion. After each pattern, we also collected from each participant a phenomenological report about the magnitude of the experienced illusory motion by obtaining a rating (on a Likert scale) of the amount of expansion of each stimulus. Specifically, we aimed to show that the pupil response is related to the magnitude of the illusory effect, that is, when the central region is black, the pupil will show a dilation response, from an equiluminant baseline, that is proportional to the subjective degree of expansion. The reason for expecting a pupillary dilation is that the black hole will evoke a sensation of a gradual loss of light, like entering a dark space. In contrast, central light patterns of different colors (e.g., green, magenta, and also white) will suggest an expansion of light, as when moving toward a differently illuminated space. Hence, we expected to observe constrictions of the pupil, from an equiluminant baseline, to the colored central regions and that these pupillary changes will also be proportional to the subjective degree of expansion.

However, considerations about the ecology of light lead us also to expect greater subjective expansions when the central region appears black than any other color. That is, when an observer moves into a dark place, this is invariably associated to the experience of an absence of light and necessarily of color. Any other scenario involving shifts in the spectrum between colored holes and surrounding colored or dark fields cannot be linked to the same type of ecological regularity or statistical learning. Hence, we predicted that both the magnitude of perceived expansion and the concomitant pupil response should be stronger when viewing black holes than in the other instances.

## Materials and Methods

### Participants

At the University of Oslo, fifty healthy participants (31 females, ages 18–41 years, mean age = 26 years; *SD* = 4.6) with normal vision were recruited. The participants were treated according to the Declaration of BMJ (1964), and the study was approved (No. 2014/1192) by the Regional Ethical Committee of Southeast Norway. All participants ticked a box in a written informed consent form, agreeing to participate anonymously in the study. We expected a small/moderate effect size of 0.4 between the pupil size at baseline vs. when exposed to the expanding illusions; hence, an appropriate sample size is 50 participants for 80% power and an alpha level of 0.05 (Brysbaert, 2019).

### Stimuli and Apparatus

**FIGURE 3:**
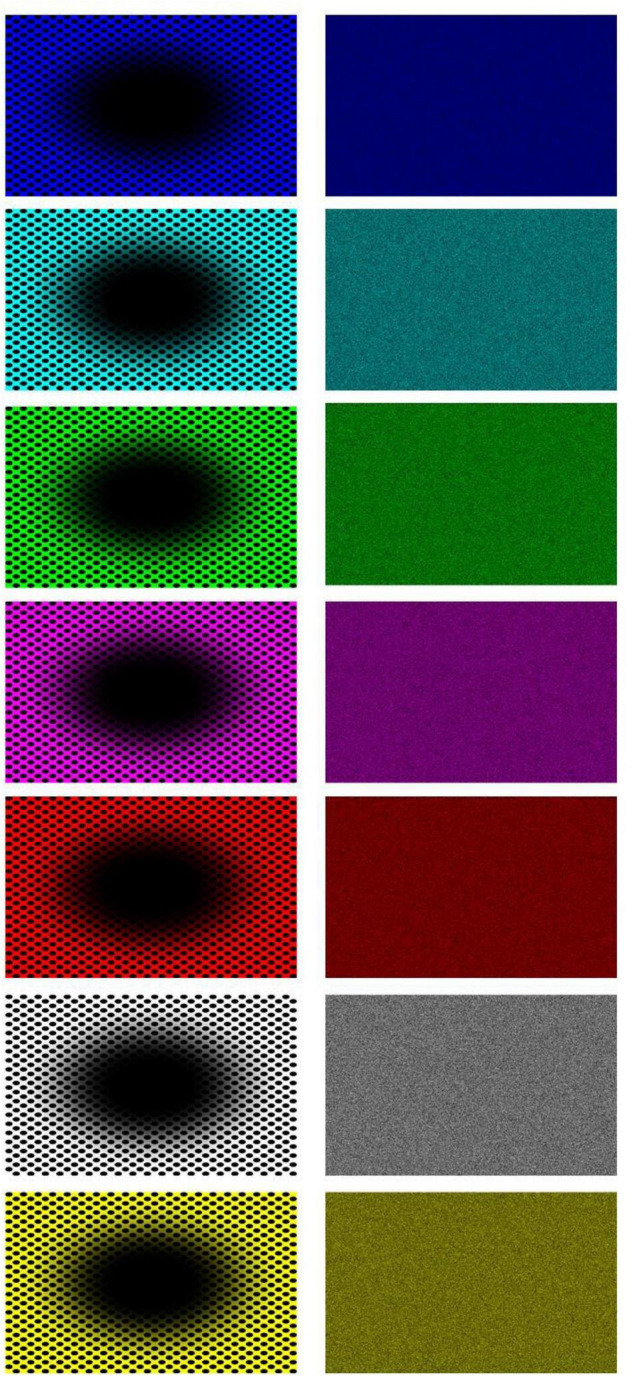
All seven “black hole” images **(left column)** and their respective baseline, scrambled pixels, images (nota bene: the expansion effect is often reduced when the images are seen in small size and on paper).

**FIGURE 4:**
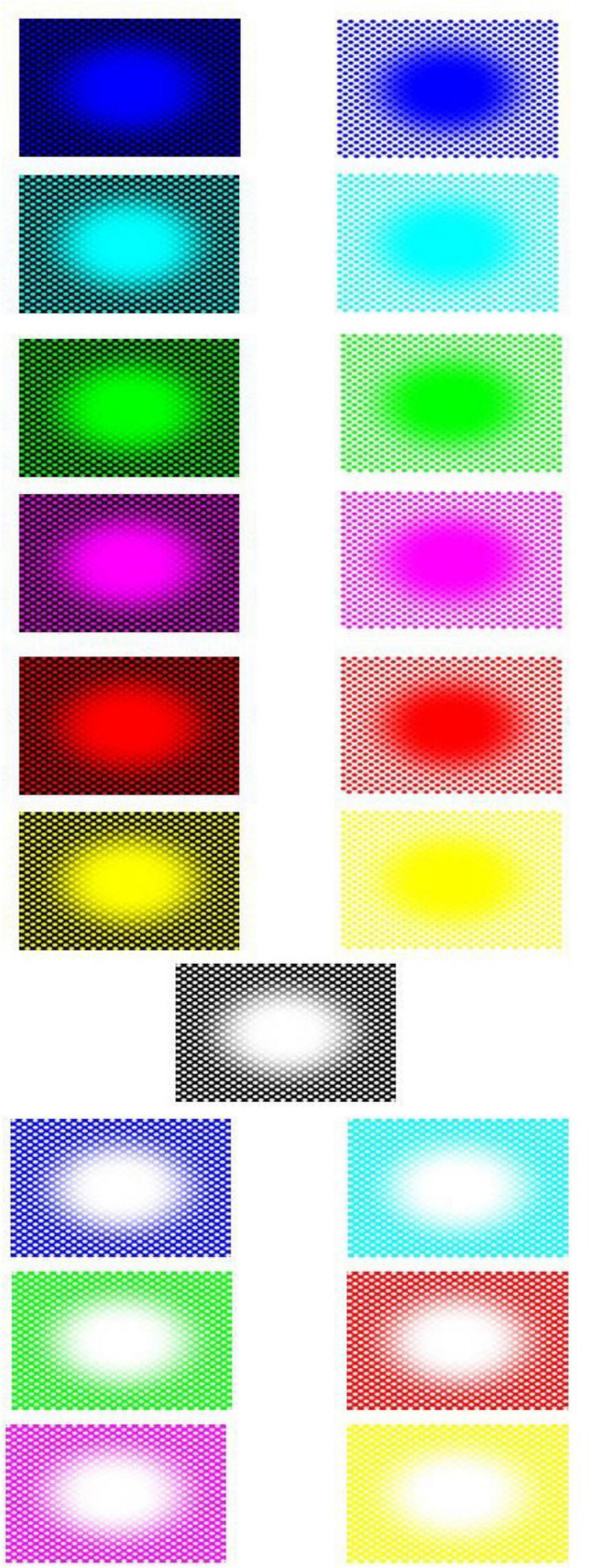
All “colored hole” images (baseline images not shown).

**FIGURE 5:**
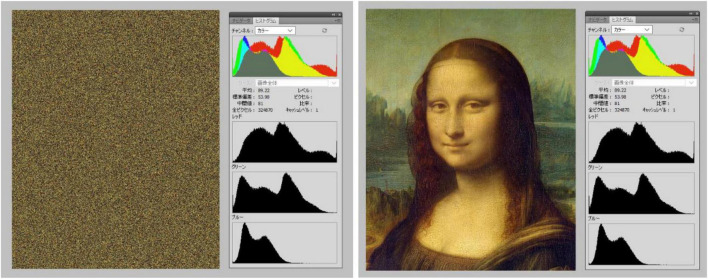
Illustration of the pixel scrambling procedure by Akiyoshi Kitaoka. The left image represents a “baseline” image for the Mona Lisa image on the right. Each panel shows a screenshot from Adobe Photoshop, with histograms showing that chromatic and luminance values of the left baseline were equal to the original on the right after scrambling.

Stimuli consisted of twenty-six patterns consisting of a central elliptical filled region on a background of equally spaced dots and twenty-six corresponding baseline images. The patterns were developed from previous examples by Fujiwara et al. (2018). We used 8 colors for either the central region, dots, or background; in HSL (i.e., Hue, Saturation, Lightness): black (170, 0, 0), blue (170, 255, 128), cyan (127, 255, 128), green (85, 255, 128), magenta (213, 255, 128), red (0, 255, 128), white (170, 0, 255), and yellow (42, 255, 128). The images with black central regions and dots were shown over one of the seven fields in color, namely, blue, cyan, green, magenta, red, white, and yellow (see Figure 3). The other 19 images showed the following combination of centers/dots vs. colored fields (refer Figure 4), namely, blue-black, blue-white, cyan-black, cyan-white, green-black, green-white, magenta-black, magenta-white, red-black, red-white, white-black, white-blue, white-cyan, white-green, white-magenta, white-red, white-yellow, yellow-black, and yellow-white. All images were generated by the last author (A.K.) using the Delphi software. The baselines were obtained by randomly scrambling one pair of pixels in each image reiteratively for 5 million times to obtain the present diffuse images (Figure 3, right column). This resulted in “snow” images, with no discernible pattern, of all randomly displaced pixels in the source image, but importantly maintaining the same average luminance as each original image. Figure 5 illustrates the results of the scrambling process using Leonardo’s *Mona Lisa* as an example.

All patterns were presented in full screen on a Dell LCD monitor with a display resolution of 1,680 × 1,050 pixels (color-calibrated using Spyder 4 Elite), while gaze and pupil diameters were monitored by an infrared SMI R.E.D. 500 (SensoMotoric Instruments, Germany), and a minimum gaze point accuracy was 0.5.

### Procedure

Participants were seated 68 cm from the screen and followed, at the start of the session, a standard 4-point calibration procedure. The head was stabilized with a chinrest aimed to reduce artifacts in pupil measurement with gaze and head movement (Sirois and Brisson, 2014). All eye-tracking data were recorded at a rate of 60 Hz. After calibration, participants began a practice block of 3 trials. All pupil measurements (including baselines) consisted of a sequence of screens, each lasting 8 s. All participants were instructed to simply look at image on the screen and avoid closing the eyes during each stimulus presentation.

## Results

We extracted expansion ratings and gaze and pupil data in each trial and for each participant using the BeGaze software (SMI). For some analyses, data were averaged across images to compare the black expanding holes to the others with no black central region, hereafter labeled “colored” (also including images with white holes).

Pupil data were based on pupil diameters (in mm) during fixations only, as computed by BeGaze, effectively excluding possible artifacts due to blinks or saccades, which can be processed and analyzed separately. No trial was excluded since the tracking ratio was above 90% in all trials. For each image, we averaged pupil diameters across fixations during each 8 s period. We subtracted the averaged pupil diameter when looking at each baseline array from that to each corresponding expanding hole stimulus. This corrected measure expresses diameter as the mean change (in mm) from baseline, with positive values indicating a dilation and negative values indicating a constriction. In addition, we also obtained standardized pupil z-scores for each expanding hole and for each participant by subtracting mean pupillary change from an individual’s mean pupillary change and then dividing by the standard deviation for each expanding hole.

### Subjective Expansion

**FIGURE 6:**
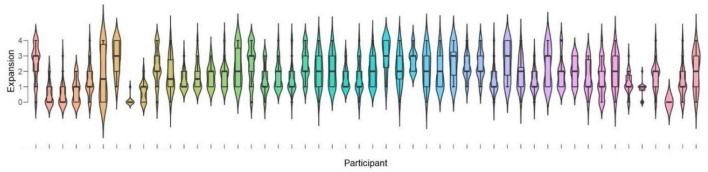
Boxplots of magnitude of expansion as rated by each participant for all “expanding holes.”

We found that participants varied considerably in their perceptions of subjective expansion. As visible in the plots in Figure 6, seven out of fifty participants subjectively experienced the central regions or “holes” as having less or no expansion (i.e., their mean expansion ratings were between 1 and 0, or “little expansion” and “none”). Interestingly, when considering non-black holes only, the number of non-susceptible individuals increased to 10. In other words, this illusion varied in strength across individuals, and only about 80% of the whole sample clearly experienced illusory motion.

**FIGURE 7:**
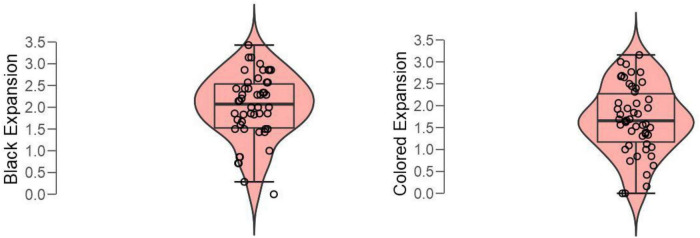
Boxplots of averaged illusory expansions with black vs. colored holes (white included).

A paired *t*-test of mean subjective expansions to black vs. colored expanding holes revealed a significant difference (Figure 7); *t*(49) = 3.05, *p* = 0.004, Cohen’s *d* = 0.432.

**FIGURE 8:**
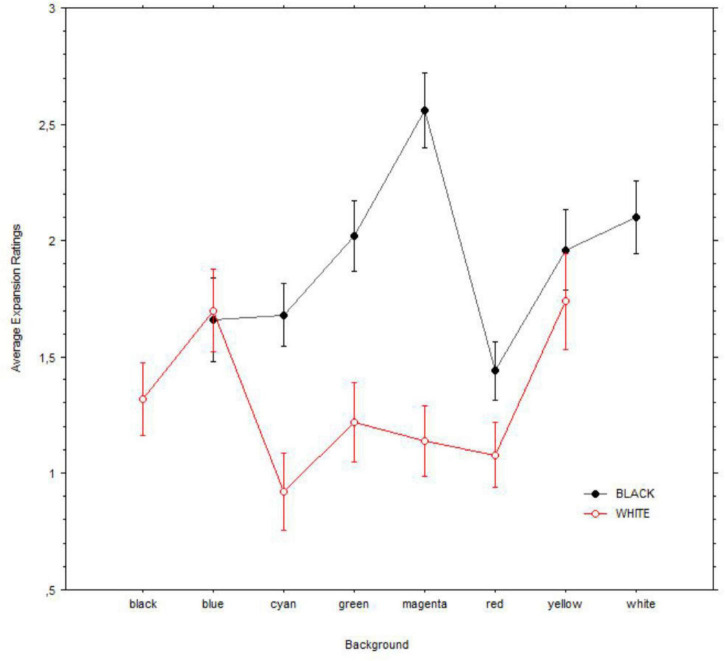
Mean illusory expansions of black (i.e., top) vs. white (i.e., bottom) holes in relation to backgrounds. Error bars are of 95% confidence intervals.

Because both the black and the white holes were tested against a background with each of the other colors, we run two separate ANOVAs on the black or white hole images with expansion (0 = none; 4 = a lot) as the dependent variable and background (i.e., blue, cyan, green, magenta, red, yellow, and white/black) as a factor. Both analyses revealed significant effect of background; black: [*F*(6, 343) = 5.5, *p* < 0.001]; white: [*F*(6, 343) = 4.8, *P* = 0.003]. Figure 8 illustrates the changes in expansion for each hole and background combination. *Post hoc* comparisons with Bonferroni correction showed that only two background colors were significantly associated with changes in expansion of black/white holes (for black: magenta vs. both blue and red; p_bonf_ < 0.001 and p_bonf_ = 0.001; for white: both blue and yellow vs. cyan; p_bonf_ = 0.025 and p_bonf_ = 0.014).

Since a common report from observers is that the illusory motion is enhanced by freely moving the eyes, we assessed the possibility that rates of gaze shifts may be proportionally related to the magnitude of the illusion and that observers who were not susceptible moved their eyes less than those who experienced subjective expansion. We found no support for these ideas since there was no relation between saccade frequency (counts per second) and ratings of expansion, *r* = 0.009, or the “dispersion” of eye position during fixations (indexing microsaccades), *r* = 0.003. Moreover, the observers who showed no susceptibility (ratings < 1) did not show significantly fewer saccades than those who were susceptible, *t*(48) = 0.48, *P* = 0.63.

### Pupil Changes

**FIGURE 9:**
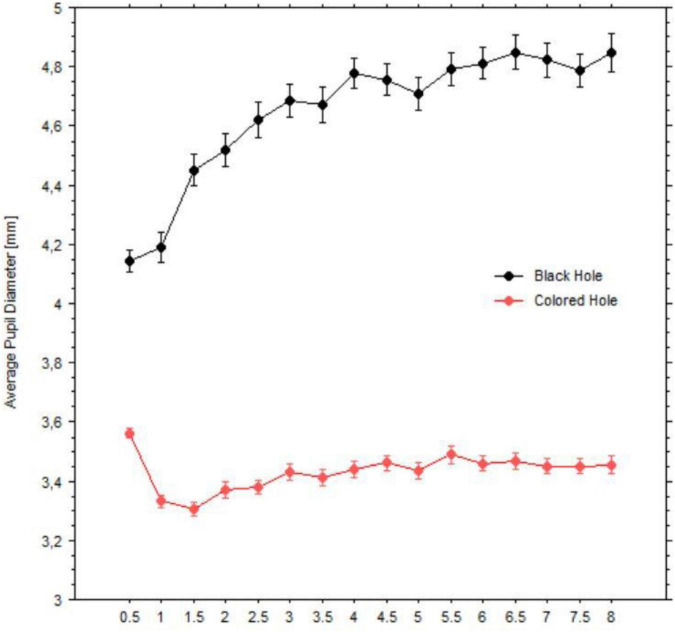
Average pupil diameters (mm) over time windows (half second) for black holes (indicated in black circles) vs. colored holes (indicated in red circles). Bars are standard errors.

Pupil diameters during fixations were averaged within each half second period, yielding sixteen epochs. Figure 9 shows changes in the pupil size in millimeters, grouped by black vs. colored holes (including white). Pupil diameters dilated over time and in a monotonic fashion when viewing the black holes; on the other hand, pupil diameters initially constricted when viewing the colored holes but showed less change afterward. The latter occurred also when viewing the baseline images of all patterns (black holes’ baselines: mean = 3.70 mm, *SD* = 0.69; colored holes’ baselines: mean = 3.98 mm, *SD* = 0.74).

**FIGURE 10:**
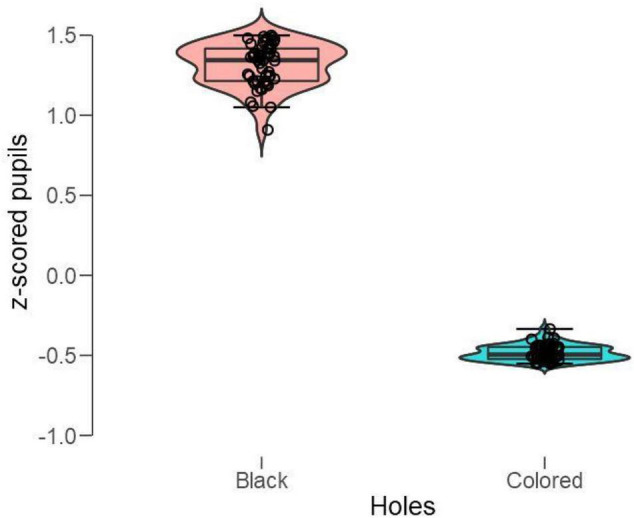
Box plots of mean standardized (z-scored) pupillary changes with black vs. colored holes (white included). Positive values indicate dilations, and negative values indicate the constrictions from baselines.

To analyze pupillary changes, we computed baseline-corrected and z-scored pupillary changes for each pattern and participant and applied a paired *t*-test to the grouped means when looking at the black holes vs. the colored holes. As shown in Figure 10, the black holes evoked strong pupillary dilations, whereas the colored holes caused the pupil to constrict; *t*(49) = 69.2, *p* < 0.001 (Cohen’s d = 9.8).

Given that the central regions of the black and colored holes are necessarily dark or bright, respectively, in relation to the baselines, it is likely that a good portion of the pupil responses simply reflect differences in luminance and contrast of this region centered on the screen in relation to the background. To assess this possibility, we performed a multiple regression analysis on the pupil *z*-scores as the dependent variable and with brightness of the central region or “hole” (or the *L*-value in HSL) and contrast (i.e., hole’s L minus background’s L/average brightness of the whole image) as the independent variables. This analysis revealed that the hole brightness significantly explained about 64% of the pupil responses, standardized coefficient = 0.81, *t* = 6.16, *p* < 0.0001. However, contrast had no significant relation to the pupil change, standardized coefficient = 0.01, *t* = 0.14, *p* = 0.89. Interestingly, an analogous multiple regression analysis with the subjective expansion ratings as the dependent variable shows no significant relation with both hole’s brightness, standardized coefficient = 0.39, *t* = 1.94, *p* = 0.065, or its contrast’s, standardized coefficient = 0.04, *t* = 0.21, *p* = 0.84. Since a sizable portion of pupillary changes remains unexplained by either brightness or contrast, it is possible that the pupillary response also relates to the conscious perception of expansion, which is what we examined in the following section.

### Relation Between Subjective Expansion and Pupil Change

Since, as shown above, the pupil dilated to black holes but constricted to the colored holes, we analyzed separately the two types by computing the Kendall correlations between the ratings of expansions for each pattern and the corresponding mean standardized (*z*-scored) pupillary changes of each participant. Kendall correlations are the (non-parametric) alternative to Pearson’s correlation and the best alternative to Spearman correlation with a small sample size and when a variable is **ordinal (e.g.,** non-numeric concepts like “little,” “a lot,” etc.; Schaeffer and Levitt, 1956) and the other is **continuous** (e.g., pupil diameters, which are nearly normally distributed, Mathôt et al., 2018, and linearly scaled in relation to baselines, Reilly et al., 2019).

For black holes, pupillary changes were positively related to the magnitude of subjective expansion; Kendall’s Tau coefficient = 0.1, *p* = 0.006. On the other hand, the same analysis with the colored holes showed no significant relationship; Kendall’s Tau coefficient = −0.005, *p* = 0.78.

To capture the relation between the growth of the pupil and subjective expansions for each pattern, we additionally computed simple regression analyses of each rating of subjective expansion for each hole pattern as the regressor and the corresponding *z*-scored pupillary change as the dependent variable. These yielded 26 regression functions, each obtaining the regression slope or beta coefficient for each pattern, expressing the magnitude or rate of change of the pupils in relation to the subjective expansions. We analyzed separately the two types by computing Kendall correlations between the obtained slopes’ coefficients (or betas) for each hole pattern (black holes: *N* = 7; colored holes: *N* = 19) and the subjective expansions, in this case, averaged over all participants.

**FIGURE 11:**
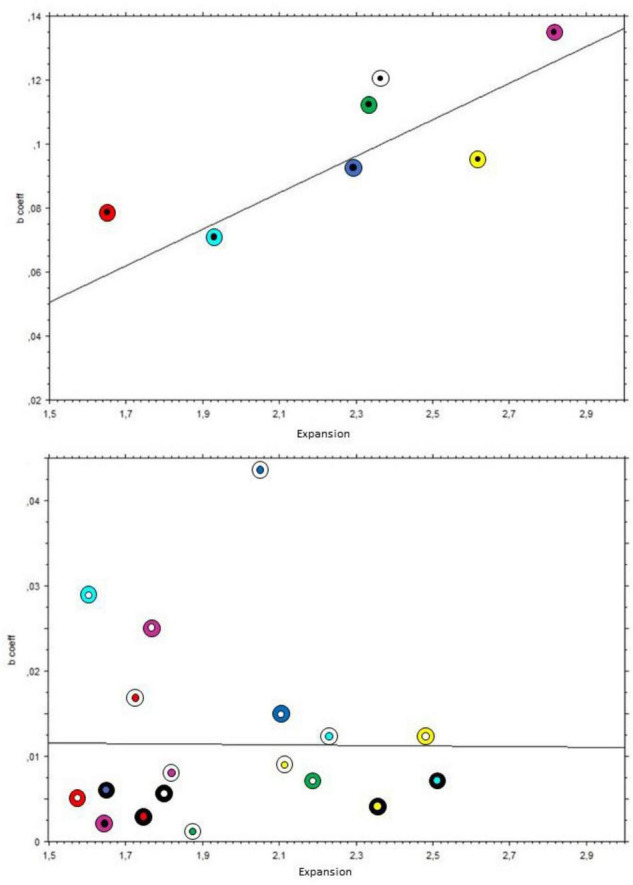
Scatterplot of slopes of pupillary changes over time of each pattern on the Y-axis, with mean subjective expansions on the X-axis. The black lines indicate the linear interpolation. Inner circles indicate the holes’ colors, and outer rings indicate the backgrounds’ colors. **Top panel:** black holes; **bottom panel:** colored holes.

The analysis on black holes revealed a moderately strong relationship between expansion and the rate of dilation of the pupil; Kendall’s Tau coefficient = 0.71, *p* = 0.02. On the other hand, the same analysis with the colored holes showed no significant relationship; Tau = 0.076, *p* = 0.65. This was also the case when considering only the patterns with white holes (*N* = 7); Tau = 0.048, *p* = 0.88. Figure 11 illustrates the positive relation between the mean subjective expansions and pupil growth rate for pupils with black holes and the lack of it with the colored holes (nota bene: The scale of the Y-axis is different in each panel, since slopes of colored holes were below the range of the black holes).

## Discussion

We collected subjective reports of illusory expansion to the pupillary adjustments while viewing the patterns shown in Figures 3, 4 and found that the reported magnitudes of illusory expansion were related to the magnitude of pupillary dilation (Figure 11). This applied to the “black holes,” but there was no significant relation between these measurements for the “colored holes” (white holes as well). This constellation of positive and negative findings is consistent with our hypotheses that, when the central region is black, the pupils adjust proportionally to the motion illusion to prepare for a change in luminance.

We based this expectation on the idea that the illusory pattern contains optical cues like a circular smear or shadow gradient of the central black hole that evokes an impression of optic flow, as if the observer were heading forward into place where light is absent or a dark hole. The illusion of a virtual expanding darkness is experienced at the cost of veridicality, since the observer is not moving forward into a real space, but both the illusory motion and the preparatory pupil adjustments are useful according to the “perceiving-the-present” account of illusions (Changizi et al., 2008), since the physiological adjustments of retinal illumination reflect how the visual world is represented in the visual cortex and suggests optimal adaptation (the next moment) according to ecological regularities.

As expected, we also found that the pupil constricted to the colored patterns in relation to an equiluminant baseline (Figure 10). However, we had also reasoned that the different colors (e.g., blue, yellow, and also white) can only suggest moving toward differently illuminated spaces, where vision is likely to remain possible. Hence, in terms of ecological predictive behavior, these conditions seem less likely to involve a risk than navigating into a completely darkened environment, where visibility is incapacitated. Hence, these negative results seem also consistent with the “perceiving-the-present” account, and we did expect that the magnitude of perceived expansion would be weaker, as well as concomitant constrictions being not related to subjective expansions. In other words, we suggest that these scenarios may need less compensatory constrictive adjustments. Instead, the case of brightness illusion like Asahi (in Figure 2) seems different because the converging gradients provide strong cues to increasing luminosity in a light source, which can be linked to the ecological likelihood of being dazzled (e.g., by the sun).

Our analyses also confirmed that, although differences in luminance across patterns did account for strength of the pupillary adjustments to different hole patterns, a portion of pupillary changes remained unexplained by either pixel brightness of the holes or their contrast with the background. We suggested that the measured pupillary responses were also related to the conscious perception of expansion and reflected oculomotor adjustments to the corresponding neural representations of the patterns, as previously suggested for brightness illusions (Laeng and Endestad, 2012).

We also found, based on the phenomenological reports when rating expansion, that a minority of observers appeared to be not susceptible to the present illusions (Figure 6). It remains unclear what could be the reasons behind these individual differences. Based on the common report that the illusion of motion may be enhanced by eye movements (e.g., Murakami et al., 2006; Troncoso et al., 2008; Zanker et al., 2010; Kapoula et al., 2015), we also tested this possibility by relating saccade rates to subjective expansions, without finding support for an increase in the susceptibility to the illusory motion with increasing mean rates of gaze shifts or that participants resilient to illusory motion would make fewer gaze shifts. Perhaps, these observers do not experience the patterns as being holes, as well as the impression of optic flow, but they may perceive them as expanding “ink splashes.” Unfortunately, we did not collect reports about how observers may have interpreted the central regions, although we can expect that people may not be aware of the assumptions made by the perceptual system about different forms of illumination (as for #TheDress illusion: e.g., Brainard and Hulbert, 2015; Wallisch, 2017).

We note that this account is based on the account by Purves et al. (2004, 2014) and Changizi (2009). However, Hering (1964) might have given a different explanation, based on the idea that contrast and assimilation mechanisms of black-white opponent processes have different spatial extents. These processes could generate similar effects independent of learning or of ecological constraints, so that they would be initially out of balance in the “white hole” cases, whereas in the case of the “black hole,” the contrast effect would prevail in time over the assimilation effect.

Finally, we found that color combinations of hole and background yielded a similar phenomenology, and most patterns did not differ in their effectiveness in evoking illusory motion (Figure 8). However, there were some exceptions: the black hole expanded the most with a magenta background in relation to both blue and red, whereas for the white holes, the maximum constriction occurred on a cyan background, compared with that of both blue and yellow (Figure 8). The magenta-related effect was unexpected, and it seems difficult to relate the strong expansion of the black hole within magenta to the ecology of light since the color is not part of the visible spectrum of light. However, magenta is associated with the perception of spectral power distributions concentrated mostly in two bands, namely, the longer wavelength reddish components and the shorter wavelength bluish components. Both spectral distributions are easily associated with the common “spectral diet” of the sky’s colors (cf. Webler et al., 2019), and it is possible that they may play a specific role in the experience of a darkening of illumination. Similarly, we surmise that the distribution of gradients that are encountered ecologically when experiencing glare may strengthen the sensitivity for cyan, particularly compared with yellow. Moreover, the Helmholtz-Kohlrausch effect (Corney et al., 2009) is stronger with blueish patches, and this effect could be in part behind the constrictive effect on the pupil of blue gradients (Suzuki et al., 2019), especially when these are present in the upper visual field (Thurman et al., 2021), also consistent with the natural ecology of sunlight.

## Data Availability Statement

The raw data supporting the conclusions of this article will be made available by the authors, without undue reservation.

## Ethics Statement

The studies involving human participants were reviewed and approved by the Departmental IRB (ref. no: 2088238) at, Department of Psychology, University of Oslo. The patients/participants provided their written informed consent to participate in this study.

## Author Contributions

BL, SN, and AK conceived and designed the study. AK created the visual stimuli. BL performed the statistical analyses and wrote the first draft of the manuscript. SN and AK edited the parts of the manuscript. All authors contributed to the manuscript revision, read, and approved the submitted manuscript.

## Conflict of Interest

The authors declare that the research was conducted in the absence of any commercial or financial relationships that could be construed as a potential conflict of interest.

## Publisher’s Note

All claims expressed in this article are solely those of the authors and do not necessarily represent those of their affiliated organizations, or those of the publisher, the editors and the reviewers. Any product that may be evaluated in this article, or claim that may be made by its manufacturer, is not guaranteed or endorsed by the publisher.

## Footnotes

1
http://www.ritsumei.ac.jp/~akitaoka/light-e.html
